# Preliminary Investigation of Pharmacist-Delivered, Direct-to-Provider Interventions to Reduce Co-Prescribing of Opioids and Benzodiazepines among a Medicare Population

**DOI:** 10.3390/pharmacy8010025

**Published:** 2020-02-21

**Authors:** Jennifer M. Bingham, Ann M. Taylor, Kevin P. Boesen, David R. Axon

**Affiliations:** 1 Tabula Rasa HealthCare, Moorestown, NJ 08057, USA; kboesen@trhc.com; 2 Pharmacy Practice and Science, University of Arizona College of Pharmacy, Tucson, AZ 85721, USA; taylor@pharmacy.arizona.edu (A.M.T.); axon@pharmacy.arizona.edu (D.R.A.)

**Keywords:** opioid, benzodiazepine, pharmacist delivered, co-prescribing

## Abstract

Co-prescribing of opioids and benzodiazepines can lead to overdoses and mortality. This retrospective study analyzed prescription claims data collected in 2016. A national medication therapy management (MTM) program conducted prescriber-based outreach interventions for patients with concurrent opioid and benzodiazepine prescriptions. The pharmacist’s direct-to-prescriber intervention was conducted following a targeted medication review. The pharmacist initiated interventions with the prescriber via facsimile to recommend discontinuation of concurrent use of these drugs. This study included 57,748 subjects who were predominantly female (67.83%) and aged ≥ 65 years (66.90%). Prescribers were most commonly located in the southern United States (46.88%). The top prescribed opioid medications were hydrocodone-acetaminophen (33.60%), tramadol (17.50%), and oxycodone-acetaminophen (15.66%). The top benzodiazepines prescribed concurrently with opioids were alprazolam (35.11%), clonazepam (21.16%), and lorazepam (20.09%). Based on the pharmacists’ recommendations, 37,990 (65.79%) resulted in a medication discontinuation (benzodiazepines 40.23%; opioids 59.77%) by the provider. There were significant differences in the proportion of opioids discontinued by subject age (*p* < 0.001) and prescriber geographical region (*p* = 0.0148). The top medications discontinued by the prescriber were hydrocodone-acetaminophen (18.86%), alprazolam (14.19%), and tramadol HCl (13.51%). This study provides initial evidence for pharmacist-supported, direct-to-prescriber programs as an effective medication safety strategy.

## 1. Introduction

Opioid use rates have doubled (from 4.1% to 9.0%) among older adults (65 years and older) in the United States (US) in recent years [1]. As a result, there are more opioid-related problems (e.g., addiction, adverse drug events), with deaths increasing by 345% between 2001 and 2016 [2]. While the recent opioid crisis has heightened awareness among the medical community, policy makers, and the general public [3,4], co-prescribing of opioids and benzodiazepines remains widespread. This is particularly concerning given the potential for associated adverse drug events, morbidity, and mortality [5]. Recently, the Centers for Disease Control and Prevention released guidelines specifically aimed at reducing overprescribing of opioids and avoiding concurrent use of opioids and benzodiazepines (potentiator drugs) [6]; potentiator drugs enhance the effects of an opioid medication when taken concurrently and may cause serious problems [7,8]. The magnitude of co-prescribing of these two drugs is an ongoing problem, and thus, it must remain a top priority in the healthcare arena.

Primary care providers (PCPs) typically are the first to intervene with patients, prescribing approximately 50% of all opioids [9]. However, many PCPs find it difficult to manage these patients given the complexity of their cases and limited knowledge regarding available resources (e.g., prescribing guidelines, pain scales) [9]. 

To this end, pharmacists are in a unique position, given their extensive clinical training in medication management and identifying medication-related problems (e.g., drug–drug interactions, dosing concerns, drug–disease interactions, adverse drug events (ADEs), monitoring concerns, and therapeutic duplication avoidance) to help address inappropriate use of opioids [10,11], and concurrent use of opioids and benzodiazepines via medication therapy management (MTM) [12]. Additionally, pharmacists play an integral role in mitigating these avoidable opioid-related drug interactions. [13] Pharmacist-delivered MTM services comprise a compendium of services designed to optimize health outcomes and reduce ADEs, including those associated with concurrent use of opioids and benzodiazepines [11,14].

Previous studies have investigated the outcomes associated with pharmacist-delivered, direct-to-provider interventions in reducing use of potentially inappropriate medications (PIMs) [10,15]. Monane et al. found that a pharmacist-delivered, telephonic (telehealth) prescriber intervention was successful in reducing the prescribing of PIMs in the elderly (e.g., Beers List) [14]. Another direct-to-provider intervention study decreased opioid utilization for chronic pain, improved adherence to guidelines, and showed a significant decrease in co-prescribing of opioids with benzodiazepines [10].

Yet, little is still known about the impact of pharmacist-delivered MTM interventions, specifically addressing co-prescribing of opioids and benzodiazepines. To address this gap, the study objectives were to describe the: (1) number of pharmacist-delivered MTM interventions to reduce co-prescribing of opioids and benzodiazepines; (2) most commonly co-prescribed opioids and benzodiazepines; and (3) number of and differences in primary care provider-accepted recommendations based on patient age, gender, and prescriber region over a one-year period. 

## 2. Materials and Methods

### 2.1. Study Design and Data Source 

This descriptive study involved a retrospective review of prescription claims data, provided by a national MTM sponsor during the period between 1 January to 31 December 2016. This project was approved by the University of Arizona Institutional Review Board. 

### 2.2. Study Participants

Subjects identified in the dataset were included if they: received a telephonic, targeted medication review (TMR), were 18 years of age or older, and taking one opioid and one benzodiazepine concurrently. 

### 2.3. Pharmacist-Delivered, Direct-to-Provider Interventions

A national MTM sponsor, housed at a dedicated, academic site, conducted automated TMRs to identify patients with concurrent prescriptions for opioids and benzodiazepines; as part of the TMR, the telepharmacist recommended discontinuing one of the co-prescribed drugs, using a standardized, call script designed to help maintain consistency in patient messaging. This automated TMR process used computerized algorithms to determine simultaneous fill dates and day supplies of medications in the two drug classes. The algorithm triggered “autoalerts” that were reviewed by pharmacy staff and authorized by a clinical telehealth pharmacist. If a provider intervention was deemed appropriate, a recommendation was delivered to the patient’s prescriber(s) via facsimile. These interventions focused on recommendations evaluating the patient’s safety risks associated with co-prescribed opioids and benzodiazepines. Pharmacy claims data were reviewed for 120 days following the intervention and deemed successful if the medication combination was not dispensed during that timeframe (e.g., discontinuation of at least one of these drug).

### 2.4. Data Collection and Analysis

Deidentified data were extracted from the prescription claims database for the 2016 calendar year. Variables of interest included: patient age and gender; provider geographic location; name and quantity of opioid medications prescribed; name and quantity of benzodiazepine medications prescribed; number and type of pharmacist-to-provider recommendations made to reduce co-prescribing of opioids and benzodiazepines; name and number of opioid and benzodiazepine medication(s) discontinued. Descriptive statistics and chi-square tests were computed using SAS v9.4 (Cary, NC, USA). An alpha value of 0.05 was selected a priori. Post-hoc chi-square tests were conducted to assess differences between regions, and a Bonferroni correction (*p* = 0.05/6 = 0.0083) was applied.

## 3. Results

### 3.1. Sample Characteristics

The study sample consisted of 57,748 subjects who were concurrently co-prescribed an opioid and benzodiazepine. Approximately two-thirds of the study sample were female (67.83%) and aged 65 years or older (66.90%). Prescribers were most commonly located in the southern region (46.88%), followed by midwestern (28.93%), northeastern (17.06%) and western (7.13%) regions of the US (See Table 1).

### 3.2. Most Commonly Co-Prescribed Medications

The top five opioid medications co-prescribed with benzodiazepines were hydrocodone-acetaminophen (*n* = 19,401, 33.60%), tramadol HCL (*n* = 10,107, 17.50%), oxycodone-acetaminophen (*n* = 9045, 15.66%), oxycodone HCL (*n* = 5349, 9.26%), and acetaminophen-codeine (*n* = 2640, 4.57%). Table A1 provides the full list of concurrently prescribed opioid medication with benzodiazepine medications. 

The top five benzodiazepine medications co-prescribed with opioids were alprazolam (*n* = 20,275, 35.11%), clonazepam (*n* = 12,222, 21.16%), lorazepam (*n* = 11,599, 20.09%), diazepam (*n* = 6988, 12.10%), and temazepam (*n* = 4233, 7.33%). Table A2 provides the full list of concurrently prescribed benzodiazepine medications with opioid medications. 

### 3.3. Recommendations Made and Accepted

For each subject, the clinical pharmacist made a direct-to-provider recommendation via facsimile to discontinue one of the medications. A total of 37,990 medications were discontinued, including opioids (*n* = 22,708, 59.77%) and benzodiazepines (*n* = 15,282, 40.23%), resulting in a 65.79% prescriber acceptance rate of pharmacists’ recommendations. Subjects whose prescribers accepted the pharmacist’s deprescribing recommendation were similar to the overall sample for females (*n* = 26,067 of 37,990, 68.6% versus *n* = 31,970 of 57,748, 67.83%) and those 65 or older (*n* = 27,063 of 37,990, 71.2% versus *n* = 38,633 of 57,748, 66.90%). Significant differences were observed in the proportion of opioids discontinued based on subject age (*p* < 0.001) and geographical region of prescriber (*p* = 0.0148) (see Table 2).

The top five medications discontinued by the prescriber in response to recommendations made by the clinical pharmacist to reduce co-prescribing of opioid and benzodiazepines were hydrocodone-acetaminophen (*n* = 7165, 18.86%), alprazolam (*n* = 5390, 14.19%), tramadol HCl (*n* = 5131, 13.51%), lorazepam (*n* = 3547 9.34%), and oxycodone-acetaminophen (*n* = 3365 8.86%). Table A3 lists the medications discontinued by prescribers to reduce co-prescribing of these two drugs.

## 4. Discussion

This descriptive study highlights commonly co-prescribed opioid and benzodiazepine medications, identified in a prescription claims database. It also provides preliminary evidence to suggest that pharmacist direct-to-provider interventions, following an MTM consultation, may help in reducing co-prescribing of opioids and benzodiazepines. 

Provider acceptance (65.79%) of pharmacist interventions, was higher than some rates previously reported [15,16,17]. Interestingly, Perera et al. found that pharmacist-delivered provider interventions communicated via facsimile had reported lower provider acceptance rates, particularly those related to guideline adherence and safety concerns [17]. An evaluation of recommendations made by MTM pharmacists, as part of an interprofessional chronic care management program, found that 37.5% (*N* = 200) of recommendations were accepted by providers [15]. Others have reported acceptance rates ranging from 42% to 60% in a community pharmacy setting and an average of 47% in a telehealth setting [16,17]. While the current study acceptance rate is higher compared to other interventions and practice settings, opportunities for improvement remain. The reasons for the somewhat higher provider acceptance rate in the current study are unknown. However, several potential explanations include subject characteristics, greater recognition of the value of pharmacist-provided MTM services, and/or the current public health priority focused on addressing the opioid epidemic.

The current study found that some providers deprescribed (either an opioid or benzodiazepine), yet the reasons for doing so are not well understood. Another study found that a direct-to-provider intervention reduced opioid dosing and significantly decreased co-prescribing of opioids with benzodiazepines [10]. However, identifying effective strategies to urge prescribers to dose reduce and/or deprescribe opioids and benzodiazepines is not without its challenges. Barriers still remain such as employing effective communication channels to ensure receipt of the pharmacist’s recommendations, prescriber knowledge regarding patients’ risk for adverse drug events, and lack of resources and institutional support for deprescribing and providing viable alternatives [18].

However, some patients still may resist deprescribing if they feel the health condition that they were using drugs to manage is critical to effective symptom management and their current treatment. In a recent study, patients expressed healthcare system factors and positive personal experiences with sedative medication usage as reasons for disputing providers’ recommendation to deprescribe while being concerned about safe alternatives [18]. Yet, numerous, viable alternatives exist rather than relying on co-prescribing opioids and benzodiazepines. For example, previous studies have found that individuals with chronic pain effectively use a variety of strategies to manage it, including both pharmacological and non-pharmacological approaches [19]. Furthermore, another study found that pharmacists with chronic pain used a mean of 13 ± 5 pharmacological and non-pharmacological management strategies, while some used as many as 31 different ones [20]. Additionally, direct-to-patient educational interventions may provide promising opportunities, especially when coupled with individualized care [18]. 

There were limitations with this study. First, it used a prescription claims database, and thus it was impossible to determine whether the medication was actually taken, if prescriptions were purchased with cash or obtained from a prescriber beyond their primary provider, or if the patient was non-compliant with prescription discontinuation and continued to take medications on-hand thereafter. Second, while the sample size was large, limited details such as a verbal or written response from the provider to the pharmacist recommendation, were available beyond whether an intervention was made and accepted. Third, this study used a sample of Medicare beneficiaries, and thus the generalizability is limited to this population. Fourth, the study had a relatively short follow-up period of 120 days, limiting its generalizability. Lastly, lack of information on the number of alerts compared to total recommendations may have contributed bias in the study regarding provider acceptance rates. Further investigation is warranted to study whether pharmacist interventions are effective in reducing co-prescription of opioids and other potentiator medications as well as exploring if deprescribing rates are maintainable over a longer study period (>4 months). 

## 5. Conclusions

This study involving prescription claims data described the characteristics of patients and the most commonly co-prescribed opioids and benzodiazepines discontinued following a pharmacist-delivered, direct-to-provider intervention. These study findings suggest that pharmacist-initiated, direct-to-prescriber communication may help reduce co-prescribing of opioids and benzodiazepines in a Medicare population. However, more work is needed, using a more comprehensive data source and analytical study design, to investigate types of pharmacist interventions made and intervention types accepted by providers. Further research is warranted to evaluate whether these same results are achievable with opioids and other potentiator medications, and to investigate this pharmacist-delivered medication therapy management program in more diverse populations and settings. 

## Figures and Tables

**Table 1:** Demographic characteristics of study subjects co-prescribed an opioid and a benzodiazepine medication (*N* = 57,748).

Characteristic		*N*	%
**Gender**			
	Female	39,170	67.83
	Male	18,578	32.17
**Age, Years**			
	<65	19,115	33.10
	65 or older	38,633	66.90
**Geographical Region of Prescriber**			
	Northeast	9853	17.06
	Midwest	16,705	28.93
	South	27,073	46.88
	West	4115	7.13

**Table 2:** Demographic characteristics of study subjects whose prescriber accepted the pharmacist recommendation to discontinue an opioid or benzodiazepine (*n* = 37,990).

Characteristic		Total *N*	Opioid *n* (%)	Benzodiazepine *n* (%)	*p* Value
**Total**			22,708 (59.77)	15,282 (40.23)	
Gender					0.2250
	Female	26,067	15,635 (59.98)	10,432 (40.02)	
	Male	11,923	7073 (59.32)	4850 (40.68)	
Age, years					<0.0010
	<65	10,927	6146 (56.25)	4781 (43.75)	
	65 or older	27,063	16,562 (61.20)	10,501 (38.80)	
Geographical region of prescriber					0.0148
	Northeast	6804	4171 (61.30)	2633 (38.70)	
	Midwest	10,965	6540 (59.64)	4425 (40.36)	
	South	17,270	10,282 (59.54)	6988 (40.46)	
	West	2948	1713 (58.11)	1235 (41.89)	

Note: Post-hoc analyses indicated a significant difference between Northeast and West regions (*p* = 0.0031). There was no difference between the other regions (*p* > 0.0083).

## Appendix A

**Table A1:** List of opioid medications co-prescribed with benzodiazepine medications (*N* = 57,748).

Opioid Name	*N*	%
ABSTRAL	1	0.00
ACETAMIN-CAFF-DIHYDROCODEINE	1	0.00
**ACETAMINOPHEN-CODEINE**	**2640**	**4.57**
ARYMO ER	2	0.00
ASCOMP WITH CODEINE	12	0.02
BELBUCA	33	0.06
BUPRENORPHINE	70	0.12
BUTALB-ACETAMINOPH-CAFF-CODEINE	35	0.06
BUTALB-CAFF-ACETAMINOPH-CODEINE	36	0.06
BUTALBITAL COMPOUND-CODEINE	34	0.06
BUTORPHANOL TARTRATE	38	0.07
BUTRANS	307	0.53
CARISOPRODOL-ASPIRIN-CODEINE	1	0.00
CODEINE SULFATE	27	0.05
CONZIP	2	0.00
DEMEROL	5	0.01
DILAUDID	22	0.04
DURAGESIC	30	0.05
EMBEDA	41	0.07
ENDOCET	290	0.50
EXALGO	8	0.01
FENTANYL	1945	3.37
FENTANYL CITRATE	7	0.01
FIORICET WITH CODEINE	2	0.00
FIORINAL WITH CODEINE #3	10	0.02
HYDROCODONEBT-HOMATROPINE MBR	1	0.00
**HYDROCODONE-ACETAMINOPHEN**	**19,401**	**33.60**
HYDROCODONE-CHLORPHENIRAMNE ER	143	0.25
HYDROCODONE-HOMATROPINE MBR	91	0.16
HYDROCODONE-IBUPROFEN	60	0.10
HYDROMET	49	0.08
HYDROMORPHONE ER	62	0.11
HYDROMORPHONE HCL	837	1.45
HYSINGLA ER	63	0.11
IBUDONE	1	0.00
KADIAN	6	0.01
LEVORPHANOL TARTRATE	8	0.01
LORCET	6	0.01
LORCET HD	9	0.02
LORCET PLUS	3	0.01
LORTAB	11	0.02
MEPERIDINE HCL	23	0.04
METHADONE HCL	501	0.87
METHADONE INTENSOL	1	0.00
METHADOSE	1	0.00
MORPHABOND ER	1	0.00
MORPHINE SULFATE	553	0.96
MORPHINE SULFATE ER	2014	3.49
MS CONTIN	3	0.01
NORCO	78	0.14
NUCYNTA	135	0.23
NUCYNTA ER	105	0.18
OPANA	6	0.01
OPANA ER	91	0.16
OXAYDO	4	0.01
**OXYCODONE HCL**	**5349**	**9.26**
OXYCODONE HCL ER	336	0.58
OXYCODONE HCL-ASPIRIN	11	0.02
OXYCODONE HCL-IBUPROFEN	1	0.00
**OXYCODONE-ACETAMINOPHEN**	**9045**	**15.66**
OXYCONTIN	1203	2.08
OXYMORPHONE HCL	82	0.14
OXYMORPHONE HCL ER	299	0.52
PERCOCET	113	0.20
PROMETHAZINE-CODEINE	408	0.71
ROXICODONE	9	0.02
SUBSYS	7	0.01
**TRAMADOL HCL**	**10,107**	**17.50**
TRAMADOL HCL ER	207	0.36
TRAMADOL HCL-ACETAMINOPHEN	424	0.73
TREZIX	1	0.00
TUSSIGON	1	0.00
TUSSIONEX	11	0.02
TUZISTRA XR	1	0.00
TYLENOL-CODEINE NO.3	32	0.06
TYLENOL-CODEINE NO.4	3	0.01
ULTRACET	12	0.02
ULTRAM	22	0.04
VICODIN	61	0.11
VICODIN ES	31	0.05
VICODIN HP	12	0.02
VITUZ	2	0.00
XARTEMIS XR	2	0.00
XODOL 7.5–300	1	0.00
XTAMPZA ER	48	0.08
ZOHYDRO ER	41	0.07

Note: Bold font indicates the top five opioid medications.

**Table A2:** List of benzodiazepine medications co-prescribed with opioid medications (*N* = 57,748).

Benzodiazepine Name	*N*	%
**ALPRAZOLAM**	**20,275**	**35.11**
ALPRAZOLAM ER	132	0.23
ALPRAZOLAM ODT	35	0.06
ALPRAZOLAM XR	75	0.13
ATIVAN	118	0.20
CHLORDIAZEPOXIDE HCL	154	0.27
CHLORDIAZEPOXIDE-AMITRIPTYLINE	40	0.07
CHLORDIAZEPOXIDE-CLIDINIUM	121	0.21
**CLONAZEPAM**	**12,222**	**21.16**
CLORAZEPATE DIPOTASSIUM	475	0.82
**DIAZEPAM**	**6988**	**12.10**
ESTAZOLAM	43	0.07
FLURAZEPAM HCL	77	0.13
HALCION	11	0.02
KLONOPIN	58	0.10
LIBRAX	54	0.09
**LORAZEPAM**	**11,599**	**20.09**
LORAZEPAM INTENSOL	14	0.02
NIRAVAM	1	0.00
ONFI	14	0.02
OXAZEPAM	150	0.26
RESTORIL	38	0.07
**TEMAZEPAM**	**4233**	**7.33**
TRANXENE T-TAB	3	0.01
TRIAZOLAM	300	0.52
VALIUM	139	0.24
XANAX	372	0.64
XANAX XR	7	0.01

Bold font indicates the top five benzodiazepine medications.

**Table A3:** Medications discontinued by prescriber following pharmacist recommendations aimed to reduce co-prescribing of opioid and benzodiazepine medications (*N* = 37,990).

Pharmacist Recommendation to Prescriber to Remove:	*N*	%
ACETAMIN-CAFF-DIHYDROCODEINE	1	0.00
ACETAMINOPHEN-CODEINE	1698	4.47
**ALPRAZOLAM**	**5390**	**14.19**
ALPRAZOLAM ER	40	0.11
ALPRAZOLAM ODT	20	0.05
ALPRAZOLAM XR	37	0.10
ASCOMP WITH CODEINE	6	0.02
ATIVAN	6	0.02
BELBUCA	9	0.02
BUPRENORPHINE	37	0.10
BUTALB-ACETAMINOPH-CAFF-CODEINE	13	0.03
BUTALB-CAFF-ACETAMINOPH-CODEINE	15	0.04
BUTALBITAL COMPOUND-CODEINE	12	0.03
BUTORPHANOL TARTRATE	14	0.04
BUTRANS	151	0.40
CARISOPRODOL-ASPIRIN-CODEINE	1	0.00
CHLORDIAZEPOXIDE HCL	61	0.16
CHLORDIAZEPOXIDE-AMITRIPTYLINE	5	0.01
CHLORDIAZEPOXIDE-CLIDINIUM	67	0.18
CLONAZEPAM	2412	6.35
CLORAZEPATE DIPOTASSIUM	147	0.39
CODEINE SULFATE	13	0.03
DIAZEPAM	2227	5.86
DILAUDID	2	0.01
DURAGESIC	3	0.01
EMBEDA	13	0.03
ENDOCET	94	0.25
ESTAZOLAM	6	0.02
EXALGO	2	0.01
FENTANYL	420	1.11
FENTANYL CITRATE	1	0.00
FIORINAL WITH CODEINE #3	1	0.00
FLURAZEPAM HCL	28	0.07
HALCION	1	0.00
**HYDROCODONE-ACETAMINOPHEN**	**7165**	**18.86**
HYDROCODONE-CHLORPHENIRAMNE ER	113	0.30
HYDROCODONE-HOMATROPINE MBR	70	0.18
HYDROCODONE-IBUPROFEN	32	0.08
HYDROMET	40	0.11
HYDROMORPHONE ER	17	0.04
HYDROMORPHONE HCL	359	0.94
HYSINGLA ER	22	0.06
IBUDONE	1	0.00
KADIAN	6	0.02
KLONOPIN	8	0.02
LEVORPHANOL TARTRATE	3	0.01
LIBRAX	20	0.05
**LORAZEPAM**	**3547**	**9.34**
LORAZEPAM INTENSOL	12	0.03
LORCET	3	0.01
LORCET HD	7	0.02
LORCET PLUS	2	0.01
LORTAB	2	0.01
MEPERIDINE HCL	10	0.03
METHADONE HCL	97	0.26
METHADONE INTENSOL	1	0.00
MORPHINE SULFATE	235	0.62
MORPHINE SULFATE ER	446	1.17
MS CONTIN	1	0.00
NORCO	10	0.03
NUCYNTA	60	0.16
NUCYNTA ER	43	0.11
OPANA	5	0.01
OPANA ER	60	0.16
OXAYDO	1	0.00
OXAZEPAM	27	0.07
OXYCODONE HCL	1604	4.22
OXYCODONE HCL ER	162	0.43
OXYCODONE HCL-ASPIRIN	4	0.01
OXYCODONE HCL-IBUPROFEN	1	0.00
**OXYCODONE-ACETAMINOPHEN**	**3365**	**8.86**
OXYCONTIN	261	0.69
OXYMORPHONE HCL	23	0.06
OXYMORPHONE HCL ER	82	0.22
PERCOCET	18	0.05
PROMETHAZINE-CODEINE	323	0.85
RESTORIL	3	0.01
SUBSYS	1	0.00
TEMAZEPAM	1095	2.88
**TRAMADOL HCL**	**5131**	**13.51**
TRAMADOL HCL ER	65	0.17
TRAMADOL HCL-ACETAMINOPHEN	235	0.62
TREZIX	1	0.00
TRIAZOLAM	63	0.17
TUSSIONEX	2	0.01
TUZISTRA XR	1	0.00
TYLENOL-CODEINE NO.3	3	0.01
ULTRACET	4	0.01
ULTRAM	6	0.02
VALIUM	22	0.06
VICODIN	43	0.11
VICODIN ES	16	0.04
VICODIN HP	8	0.02
XANAX	34	0.09
XANAX XR	4	0.01
XARTEMIS XR	2	0.01
XODOL 7.5-300	1	0.00
XTAMPZA ER	21	0.06
ZOHYDRO ER	8	0.02

Note: Bold font indicates the top five medications removed by the prescriber in response to recommendations made by the pharmacist to reduce co-prescribing of opioid and benzodiazepine medications.
